# Outcomes of stereotactic body radiotherapy versus lobectomy for stage I non-small cell lung cancer: a propensity score matching analysis

**DOI:** 10.1186/s12890-019-0858-y

**Published:** 2019-05-22

**Authors:** Qingren Lin, Xiaojiang Sun, Ning Zhou, Zhun Wang, Yaping Xu, Yuezhen Wang

**Affiliations:** 1 Department of Radiation Oncology, Zhejiang Cancer Hospital, 1 Banshan dong Road, Hangzhou, People’s Republic of China; 2 Comprehensive Cancer Center, Charité Universitätsmedizin Berlin, Charitéplatz 1, D-10117 Berlin, Germany

**Keywords:** Non-small cell lung cancer, Stereotactic body radiotherapy, Lobectomy, Propensity score match

## Abstract

**Background:**

Lobectomy is the standard treatment for patients with stage I non-small cell lung cancer (NSCLC). Recent studies have shown promising results of stereotactic body radiation therapy (SBRT) in these patients. We retrospectively compared the outcomes of lobectomy and SBRT in these patients from our therapeutic center.

**Methods:**

Patients who underwent lobectomy or SBRT for clinical T1–2a (T size≤5 cm), N0 M0, NSCLC between December 2011 and August 2016 were reviewed. Patient characteristics, treatment-related outcomes and toxicities were analyzed. Propensity score matching (PSM) was performed to improve comparability between the two groups.

**Results:**

Median follow-up period in the lobectomy (*n* = 246) and SBRT (*n* = 70) group was 31.4 months and 24.9 months, respectively. Three-year local recurrence-free survival (LRFS) was comparable in the two groups (97% vs. 91.7%, respectively; *P* = 0.768). Recurrence-free survival (RFS) at 3-year in the lobectomy and SBRT groups was 85.4 and 69.5%, respectively (*P* = 0.014). Three-year overall survival (OS) after lobectomy and SBRT was 88.2 and 79.7%, respectively (*P* = 0.027), while 3-year cancer-specific survival (CSS) was 91.3 and 82.5% (*P* = 0.022). After PSM (45 matched patients in each group), there was no significant between-group difference with respect to 3-year LRFS (89.6% vs. 87.5%, *P* = 0.635), RFS (77.6% vs. 67.3%, *P* = 0.446), OS (78.5% vs. 79.5%, *P* = 0.915) or CSS (86.4 and 79.5%, *P* = 0.551). In matched subgroup, 30-day mortality after lobectomy was 2.2%, and no treatment-related death occurred after SBRT.

**Conclusions:**

Treatment-related outcomes of SBRT and lobectomy were comparable. SBRT was well tolerated and had a very low toxicity profile in our study. SBRT is a promising alternative treatment option for stage I NSCLC patients. This study indicates that matching these disparate cohorts of patients is challenging. Clinical trials are essential to define the indications and relative efficacy of lobectomy and SBRT in a selected population.

## Background

Due to the continuing growth of the geriatric population and the application of computed tomography (CT) screening, more patients with stage I non-small cell lung cancer (NSCLC) were diagnosed and underwent therapy in recent years [1]. Lobectomy with mediastinal lymph node dissection or sampling is the standard therapy for patients with medically operable stage I NSCLC. However, the available treatment options are often limited in geriatric patients, those with comorbidity and not willing or unable to tolerate surgery [2].

Over the past decade, the use of conventionally fractionated radiotherapy has increasingly been replaced by stereotactic body radiation therapy (SBRT) for patients who are not willing or unable to undergo surgery. Results of retrospective and phase II prospective studies have shown that local recurrence-free survival (LRFS) achieved with SBRT is approximately 90%, which is similar to that achieved with surgery in patients with operable stage I NSCLC [3–5]. Besides, the performance of SBRT was shown to be effective even in geriatric patients with G8 scores (a screening tool used to predict functional decline and OS in elderly patients with cancer) ≥13, and to improve the long-term survival of these patients [6]. However, phase III randomized studies (including the ROSEL, the STARS, and the ACOSOG Z4099 trials) that sought to compare SBRT with surgery in patients with stage I NSCLC failed to reach their accrual goal and were terminated.

With increasing use of SBRT, the treatment model and patterns of care for patients with stage I NSCLC have witnessed a rapid change. Whether outcomes of SBRT in patients with stage I NSCLC are equivalent to those of surgery will continue to be debated in the absence of robust data from randomized trials. In geriatric patients and those with other risks for surgery, there is a need to evaluate the choice of treatment in Stage I NSCLC. In this study, we retrospectively compared the outcomes of SBRT with those of lobectomy in a cohort of patients with stage I NSCLC in our therapeutic center.

## Materials and methods

### Patient eligibility

Patients with clinical stage I NSCLC who were treated with lobectomy or SBRT between December 2011 and August 2016 at the Zhejiang Cancer Hospital were eligible for inclusion. The study protocol was approved by the medical ethics committee at the Zhejiang Cancer Hospital (No. IRB-2018-134). The inclusion criteria were: pathologically or clinically diagnosed NSCLC; confirmed as clinical Stage I (T1–2aN0M0) according to the TNM classification (UICC 7th edition, 2009); all patients accepted SBRT or lobectomy. Patients for whom histopathology results were not available were clinically confirmed as NSCLC based on the malignant growth characteristics of the primary lesion with an increase in the longest axis on repeat CT examination and hyper-metabolic activity on fluorodeoxyglucose-positron emission tomography (FDG-PET) [7]. Multidisciplinary consultations were made before SBRT for patients without histopathology results.

### Treatment

In the SBRT group, 4-dimensional CT scan was used to record tumor motion related to respiration in all patients. All patients were simulated in the condition of free breathing. The gross tumor volume (GTV) was contoured mainly based on CT and FDG-PET CT. The internal target volume (ITV) was created from the GTV and determined by the tumor motion. The planning target volume (PTV) was defined as the ITV plus a 5-mm margin and 1.0 cm in the longitudinal plane (craniocaudal). The treatment plan was generated according to isocentric or non-isocentric inverse-planning algorithm on the Raystation 4,5,1 system. Adequate target coverage of 100% of the ITV and > 95% of PTV was to be achieved with the highest isodose line of the prescribed dose.

In the lobectomy group, the mode of thoracic surgery (video-assisted or thoracotomy) was determined based on multidisciplinary consultation prior to the procedure.

### Follow-up

Patients underwent follow-up at every 3 months in the first 2 years after treatment, and every 6 months in the 3rd year. CT scan was done routinely during follow-up. In case of suspected disease relapse, FDG-PET scans were repeated for confirmation.

Local recurrence (LR) was defined as a recurrence or development of a new lesion adjacent to PTV in the same lobe, which corresponded to hyper-metabolic areas on FDG-PET CT and/or was confirmed histologically after SBRT, or as recurrence on the resection margin after lobectomy. Regional recurrence (RR) was defined as a failure in the hilum, mediastinum or supraclavicular fossa. Distant metastasis (DM) was defined as a recurrence in a different lobe or in an extrathoracic organ. Recurrence-free survival (RFS) was calculated from the first day of diagnosis to the date of tumor recurrence (including LR, RR and DM). OS and cancer-specific survival (CSS) were calculated from the first day of diagnosis to the death or the most recent follow-up.

### Evaluation of treatment associated toxicity

Treatment-related toxicity was recorded and evaluated based on the National Cancer Institute’s Common Terminology Criteria for Adverse Events (NCI CTCAE, version 4.0). Treatment-related mortality was defined as death during the period of hospitalization for treatment or within 30 days of the procedure.

### Statistical analysis

Categorical data were compared using Fisher exact test; continuous data were compared using *t* test. The median follow-up duration was estimated by the reverse Kaplan-Meier method. The Kaplan–Meier method was used to analyze the time to RFS, OS, and CSS, and the between-group differences were assessed using the log-rank test. All analyses were performed using IBM SPSS 21.0 software (IBM, Inc.) with a macro to calculate the cumulative incidence competing risk. All significance tests were two tailed, with significance set at *P* less than 0.05. Propensity score matching (PSM) was used to reduce selection bias from confounding factors between the lobectomy and SBRT group. PSM accounted for age, gender, WHO performance status (PS), pulmonary function (forced expiratory volume in 1 s [FEV1] % and FEV1), and T stage. Matching was performed in a blinded manner (1:1 ratio, caliper distance = 0.005) without replacement using a semi-automated method in the *MatchIt* package (version 4.8.3.4) for R (version 3.0.1) [8].

## Results

### Patient characteristics

A total of 316 patients with clinical stage I NSCLC were included. These patients were treated with lobectomy (*n* = 246) or SBRT (*n* = 70). The baseline characteristics of the patients are shown in Table 1.

**Table 1:** Characteristics of patients in the lobectomy and SBRT groups before propensity score matching

Characteristics	Patient no., %		*P*
	Lobectomy (*n* = 246)	SBRT (*n* = 70)	
Mean age ± SD (years)	66.41 ± 7.39	71.63 ± 8.80	0.000
Gender			
Female	115 (46.7)	23 (32.9)	0.039
Male	131 (53.3)	47 (67.1)	
WHO PS			
0	124 (50.4)	20 (28.6)	0.001
1	122 (49.6)	49 (70.0)	
2	0	1 (1.4)	
T stage			
T1a	124 (50.4)	53 (48.6)	0.113
T1b	73 (29.7)	44 (40.0)	
T2a	49 (19.9)	18 (11.4)	
PFT			
FEV1 (L), mean ± SD	1.96 ± 0.47	1.48 ± 0.60	0.000
Percentage predicted			
FEV1 (%), mean ± SD	83.21 ± 16.00	68.26 ± 25.51	0.000

*PS* performance status, *PFT* Pulmonary function test, *FEV1* forced expiratory volume in 1 s, *SBRT* stereotactic body radiation therapy, *SD* standard deviation

The median age of patients who underwent lobectomy was 67 (range, 39–83) years while the median age of those who underwent SBRT was 72.5 (range, 52–88) years. Patients with SBRT showed significantly poorer PS and lower FEV1. The proportion of clinical stage T1 in the SBRT group was similar to the lobectomy group (*P* = 0.133). Before treatment, 51 (72.9%) SBRT patients and 48(19.5%) lobectomy patients had histological confirmation. Forty-five (64.3%) patients in the SBRT group and 32 (13%) patients in the lobectomy group underwent FDG-PET scan.

### Treatment

In the lobectomy cohort, video-assisted thoracic surgery was the most common operation (178/246, 72.4%), while the remaining patients underwent thoracotomy (68/246, 27.6%). Radiologically negative nodal involvement was detected as positive in 15 patients. Of these, 10 patients were upstaged to pathological N1, and 5 patients had N2 after surgery respectively, because of hilar and mediastinal lymph metastasis that was identified after lymph node sampling. Among these, 3 surgical patients received epidermal growth factor receptor tyrosine kinase inhibitors (EGFR-TKI), and one patient received adjuvant chemotherapy only; none of the patients received adjuvant radiotherapy. In addition, 20 patients were upstaged to pathologic T2 tumors. None of the patients developed a synchronous tumor in a separate lobe at the time of surgery.

In the SBRT cohort, different fractionation schemes (Table 2) were used (range, 4–10 fractions) and the total radiation dose delivered was 50–70 Gy. All the patients received sufficiently high doses, of which biologically effective dose (BED) was no less than 100 Gy. Forty-three (61.4%) patients received BED 100 Gy with total dose of 50 Gy delivered in 5 fractions, while 14 (20%) patients received BED 112.5 Gy in 4 fractions. Seven (10%) patients received BED 105 Gy with total dose 60 Gy delivered in 8 fractions, while 2 (2.8%) patients received BED 120 Gy in 6 fractions. Three (4.3%) patients received BED 115.5 Gy with total dose of 55 Gy delivered in 5 fractions. One (1.4%) patient received BED 119 Gy with total dose of 70 Gy in 10 fractions. None of the patients received adjuvant chemotherapy until disease progression.

**Table 2:** Distribution of BED, fractions and doses in SBRT group

Prescribed total dose	No. of fractions	BED	Patient no., %
50	5	100	43 (61.4)
50	4	112.5	14 (20)
55	5	115.5	3 (4.3)
60	8	105	7 (10)
60	6	120	2 (2.8)
70	10	119	1 (1.4)

*BED* biologically equivalent dose

### Survival, pattern of recurrence before PSM

**Fig. 1:**
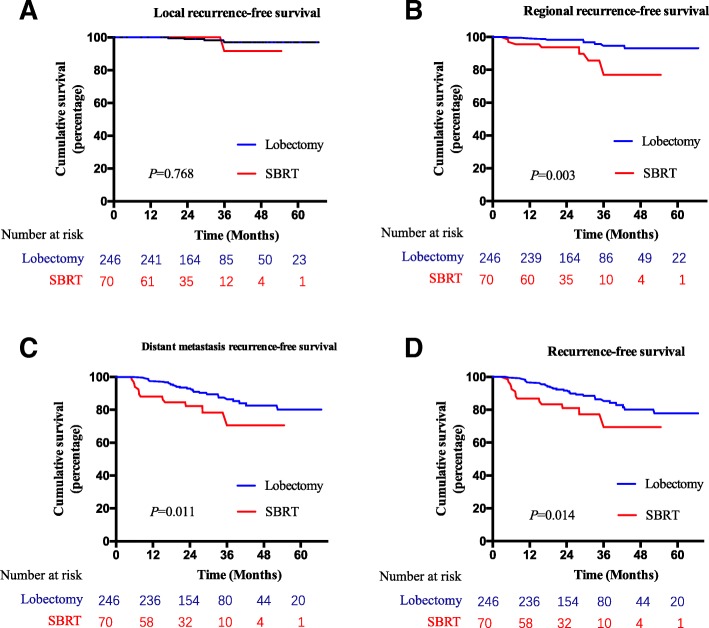
Comparison of local recurrence-free survival (**a**), regional recurrence-free survival (**b**), distant metastasis recurrence-free survival (**c**), and recurrence-free survival (**d**) of patients in lobectomy or SBRT group before propensity score matching. SBRT = stereotactic body radiation therapy

The median follow-up duration of patients in the lobectomy and SBRT groups was 31.4 (range, 0.3–66.7) months and 24.9 (range, 2.4–54.6) months, respectively. A total of 32 (13%) patients in the lobectomy group and 14 (20%) in the SBRT group developed disease recurrence within the observation time. Among these, 4 (1.6%) patients developed LR after lobectomy while 1 (1.4%) patient developed LR after SBRT; actuarial LRFS at 3 years was 97 and 91.7%, respectively (Fig. 1a). No significant between-group difference was observed with respect to LRFS (*P* = 0.768). Any new or growing lesions were closely assessed with repeat CT scan, in order to distinguish LR from fibrosis. LR was confirmed by histological examination (1 patient) or a combination of CT and FDG-PET (4 patients). Nine (3.7%) patients in the lobectomy group and 7 (10%) patients in the SBRT group developed RR during follow-up; the corresponding 3-year regional recurrence-free survival (RRFS) was 94.6 and 77%, respectively. RRFS was significantly better after lobectomy as compared to that after SBRT (*P* = 0.003) (Fig. 1b). Besides, DM was the main reason for treatment failure. Twenty-eight (11.4%) patients in the lobectomy group and 13 (18.6%) patients in the SBRT group developed DM. The lobectomy group was significantly better than SBRT group with respect to 3-year distant metastasis recurrence-free survival (DMRFS) (86.4 and 70.8%, respectively; *P* = 0.011) (Fig. 1c). Overall, a significant between-group difference was observed with respect to RFS. The 3-year RFS after lobectomy and SBRT was 85.4 and 69.5%, respectively, *P* = 0.014 (Fig. 1d).

**Fig. 2:**
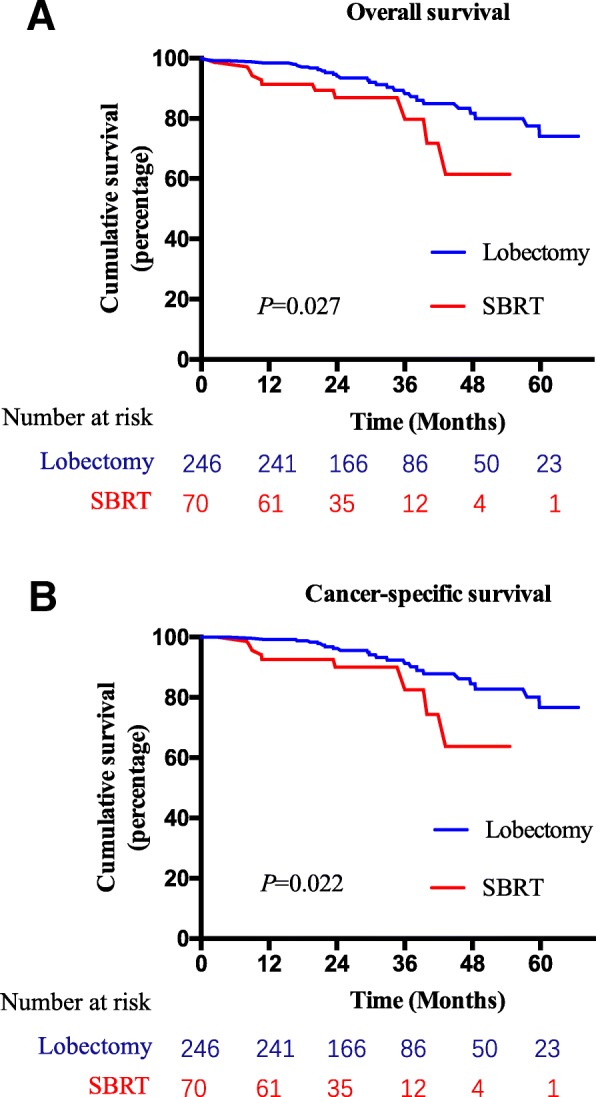
Comparison of overall survival (**a**), cancer-specific survival (**b**) of patients in lobectomy or SBRT group before propensity score matching. SBRT = stereotactic body radiation therapy

A total of 28 (11.4%) patients in the lobectomy group and 11 (15.7%) patients in the SBRT group died during the follow-up period. The survival outcomes in the lobectomy group were better than those in the SBRT group. The 3-year OS after lobectomy and SBRT was 88.2 and 79.7%, respectively (*P* = 0.027) (Fig. 2a). Among them, 22 (8.9%) patients in the lobectomy group and 9 (15.7%) patients in the SBRT group died of lung cancer. The 3-year CSS in the lobectomy group (91.3%) was significantly better than that in the SBRT group (82.5%, *P* = 0.022) (Fig. 2b). The other causes of death in the lobectomy group included pulmonary infection unrelated to surgery (1 patient), surgery-related toxicity (3 patients) and second primary cancer (2 patients). The other reasons for death in the SBRT group were second primary cancer (1 patient) and respiratory failure unrelated to treatment (1 patient).

### Toxicity before PSM

No treatment-related death was encountered in the SBRT group. Adverse events associated with SBRT included grade 1–2 pneumonitis in 27 (38.6%) patients, and grade 3 pneumonitis in 1 patient. The other mild complications in 29 (41.4%) patients are detailed in Table 3. In the lobectomy group, 3 patients died within 30 days after surgery due to post-operative pulmonary infections; 30-day mortality in the surgery group was 1.1%. One patient experienced bronchial fistula after surgery. Three patients developed hoarseness of voice after surgery. Twenty-eight (11.4%) patients experienced other grade 1–2 surgery-related complications (Table 3).

**Table 3:** Toxicity of patients in the lobectomy and SBRT groups

Toxicity	Patient no., %	
	Lobectomy (*n* = 246)	SBRT (*n* = 70)
Pneumonitis		
Grade 1–2	11 (4.8)	27 (38.6)
Grade 3	0	1 (1.4)
Grade 5	3 (1.2)	0
Bronchial fistula	1 (0.4)	0
Hoarseness	3 (1.2)	0
Chest pain	13 (5.3)	4 (5.7)
Fatigue	1 (0.4)	4 (5.7)
Esophagitis	0	1 (1.4)
Dyspnea	0	7 (10)
Cough	0	11 (15.7)

### Survival, pattern of recurrence after PSM

PSM resulted in a final cohort of 90 patients (45 lobectomy and 45 SBRT patients) who were eligible for further analysis. Patient characteristics are summarized in Table 4. The patients’ characteristics, including gender, age, T stage, pulmonary function (FEV1% and FEV1), and WHO PS were comparable between the two cohorts. The follow-up period of these two cohorts ranged from 0.3 to 64.9 months (median follow-up period: 29.5 months).

**Table 4:** Characteristics of patients in the lobectomy and SBRT groups after propensity score matching

Characteristics	Patient no., %		*P*
	Lobectomy (*n* = 45)	SBRT (*n* = 45)	
Mean age ± SD (years)	70.11 ± 8.07	69.44 ± 8.45	0.708
Gender			
Female	18 (40)	18 (40)	1
Male	27 (60)	27 (60)	
WHO PS			
0	23 (51.1)	18 (40)	0.290
1	22 (48.9)	27 (60)	
T stage			
T1a	17 (37.8)	22 (48.9)	0.426
T1b	21 (46.7)	15 (33.3)	
T2a	7 (15.6)	8 (17.8)	
PFT			
FEV1 (L), mean ± SD	1.76 ± 0.47	1.78 ± 0.52	0.864
Percentage predicted			
FEV1 (%), mean ± SD	79.82 ± 19.85	80.42 ± 19.07	0.884

*PS* performance status, *PFT* Pulmonary function test, *FEV1* forced expiratory volume in 1 s, *SBRT* stereotactic body radiation therapy, *SD* standard deviation

**Fig. 3:**
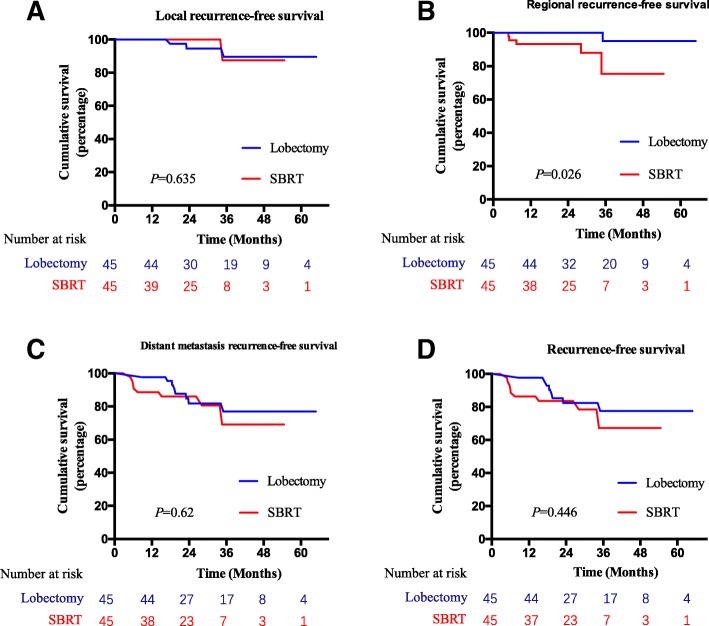
Comparison of local recurrence-free survival (**a**), regional recurrence-free survival (**b**), distant metastasis recurrence-free survival (**c**), and recurrence-free survival (**d**) of patients in lobectomy or SBRT group after propensity score matching. SBRT = stereotactic body radiation therapy

At 3 years, LRFS in the SBRT group was 87.5% (only one patient experienced LR) as compared to 89.6% (3 patients experienced LR) in the lobectomy group (*P* = 0.635) (Fig. 3a). At 3 years, 5 patients in the SBRT group experienced RR compared to 1 patient in the lobectomy group. The 3-year RRFS after lobectomy and SBRT was 95 and 75.4%, respectively. Lobectomy was still significantly better than SBRT (*P* = 0.026) (Fig. 3b). Eight patients in the SBRT group developed DM, as well as the lobectomy group. The 3-year DMRFS after lobectomy were 77 and 69.1%, respectively. The difference in DMRFS in the matched pairs became insignificant after PSM (*P* = 0.62) (Fig. 3c). There was no difference between the paired groups with respect to 3-year RFS (77.6% vs. 67.3%, *P* = 0.446) (Fig. 3d).

**Fig. 4:**
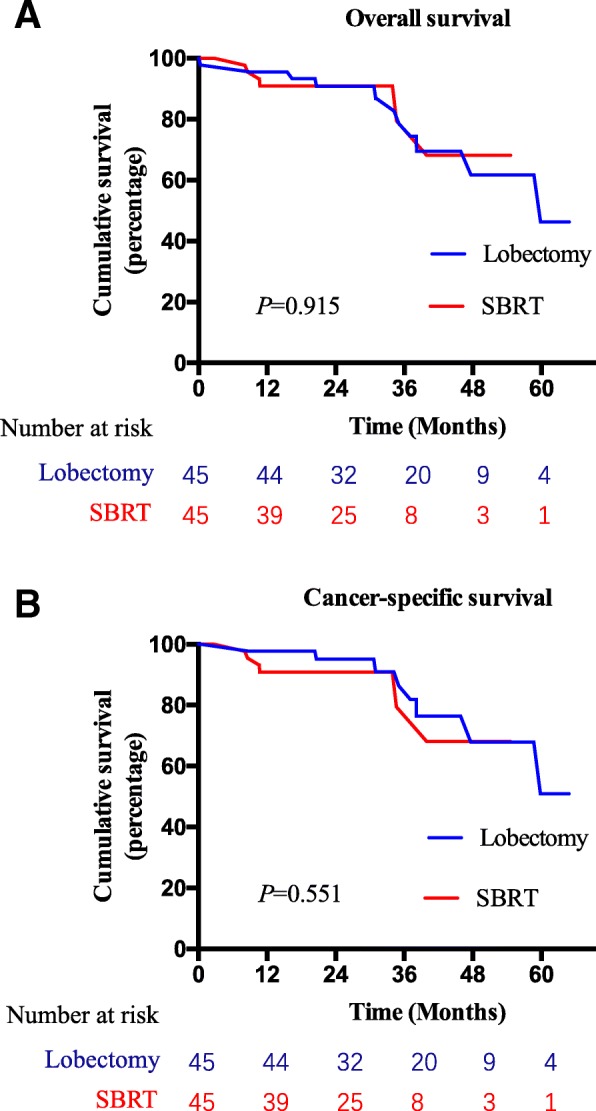
Comparison of overall survival (**a**), cancer-specific survival (**b**) of patients in lobectomy or SBRT group before propensity score matching. SBRT = stereotactic body radiation therapy

After PSM, the 3-year OS in the surgery and SBRT groups was 78.5 and 79.5%, respectively (*P* = 0.915). CSS was also similar between the two groups (86.4 and 79.5%, respectively; *P* = 0.551) (Fig. 4).

### Toxicity after PSM

In the lobectomy group, there were one death due to post-operative pulmonary infection within 30 days after surgery, and hence the 30-day mortality was 2.2%. Two patients experienced hoarseness of voice after surgery, which had an adverse impact on the quality of life. There was no death due to adverse effects in the SBRT group. During the entire follow-up period, 20 patients had mild (1–2 pneumonia) pulmonary adverse effects, which did not affect everyday life.

## Discussion

Our intent was to assess the difference between lobectomy and SBRT in stages I of NSCLC. In the past decade, SBRT has become the preferred treatment in the medically inoperable population due to high rates of local control, convenience, and suggestion of better survival compared to historic outcomes of conventional radiation. For low-risk patients with stage I NSCLC, lobectomy with mediastinal lymph node sampling/dissection is considered the standard care. However, for patients who are medically operable [9] but have a high perioperative risk, the treatment decision-making is often made subjectively on an individual basis because of the lack of evidence-based guidelines.

In this retrospective study, LRFS in the lobectomy and SBRT groups were both similar before and after PSM. In the past few decade, several retrospective and prospective studies had shown that SBRT achieved similar excellent LRFS in inoperable and operable stage I NSCLC [3, 10, 11]. In the present study, LRFS after SBRT was not inferior to that after lobectomy, before and after PSM, which is consistent with several retrospective PSM studies [9, 12]. Pooled analysis of data from two randomized trials (STARS and ROSEL) did not find any significant difference between the two treatment groups with respect to LRFS (96% vs. 100%, *P* = 0.44) [10]. Furthermore, a recent report about the 7-year follow-up after SBRT for patients with stage I NSCLC found outstanding outcomes with low local recurrence rate (8.1%) [13], which was not inferior to lobectomy.

The present study effectively used PSM to balance the covariates in the two groups, which minimized the influence of confounding variables. Before PSM, patients in the lobectomy group achieved significantly better RRFS, DMRFS, RFS, OS and CSS than SBRT. The reason might be that patients who received SBRT were older and had significantly worse PS and pulmonary function. After PSM, no statistically significant differences were identified with respect to DMRFS, RFS, OS or CSS.

In our study population, the main patterns of failure were RR and DM. There was significant between-group difference with respect to RR, while there was no significant difference with respect to DM or RFS after PSM. Firstly, this result suggests that SBRT patients may have been understaged before treatment due to the lack of invasive mediastinal and hilar staging and the lower proportion of patients who underwent FDG-PET scan in the study. Without these modes of diagnosis, the occult site is not easily detected with current imaging modalities. So PET-CT scans should be routinely performed prior to the treatment. Secondly, lobectomy with systemic mediastinal lymph node sampling/dissection showed an advantage over SBRT in terms of reduced incidence of RR. More importantly, the incidence of DM was lower in SBRT patients, which suggests that SBRT may offer benefit by targeting occult metastases, thereby leading to improved prognosis. To explain this, a study indicated the hypothesis that SBRT has unique mechanisms for improving immune function, which is mainly mediated by CD8+ T cells [14]. So, a new concept of Immuno Stereotactic Ablative Body Radiotherapy (ISABR), a combination of immunotherapy and SBRT, has been proposed and applied in several ongoing clinical trials [15].

In our study, there was no difference between SBRT and lobectomy with respect to 3-year OS and CSS. However, previous studies have yielded mixed results in this respect. In a recent Phase 2 clinical trial at the MD Anderson Cancer Center with long-term follow-up, the 7-year OS after SBRT was 47.5% [13]. Notably, a pooled analysis of two Phase III randomized trials ROSEL and STARS, suggested that 3-year OS after SBRT were better than that after lobectomy (95% vs. 79%, *P* = 0.037) [10]. In spite of many limitations in the two trials, the study seemed to provide some support for SBRT and prompted a discussion about the results. Owing to the lack of robust data from prospective studies, retrospective propensity score comparisons of the two therapies have been used to compare the therapeutic efficacy of the two methods. Retrospectively analyzed data from 19,882 patients recruited in 16 studies compared the outcomes between SBRT and surgery with PSM [16]. After PSM, the lobectomy patients had better OS than that of SBRT patients with hazard ratios of 1.61 (95% CI: 1.27–2.03) for OS and 2.14 (*P* = 0.10); however, no significant difference was observed with respect to CSS with hazard ratios of 1.35 (95% CI: 0.70–2.62). However, Shirvani et al retrospectively analyzed 10,923 elderly patients from the SEER database with PSM, and found that the OS and CSS were similar in the two groups [17]. Others have found equivalent intermediate-term OS as well [9]. However, there were several shortcomings in these studies including the definition of recurrence and heterogeneity with respect to the delivery of SBRT across different centers [11]. Therefore, no definitive conclusions can be drawn at this stage. Several clinical trials have been recently launched to answer this question. SABRTooth is a United Kingdom trial comparing SBRT and surgery in patients with peripheral early-stage NSCLC; however, it was closed to accrual recently [18]. POSTLIV is a Chinese Phase II trial comparing radical resection versus SBRT in patients with peripheral stage I NSCLC. As a member of the clinical trial, we are still enrolling patients [19]. VALOR is recruiting patients from the Veterans Affairs medical centers across the United States to compare OS between resection and SABR for both peripheral and central lesions [20].

With respect to treatment-related toxicity, the published data and our findings suggest that SBRT is associated with a low incidence of mortality and morbidity compared with lobectomy [21]. In previous studies, the majority of patients experienced asymptomatic adverse effects, including mild (grade 1–2) radiation pneumonitis (56%), and chest wall pain (22%) [13]. Lobectomy, even with minimally invasive surgical techniques, resulted in more severe complication, including postoperative pneumonia (4.3%), bronchial fistula (0.4%) and pulmonary embolism (0.5%) [22]. With more severe toxicity, patients in lobectomy group often died within 30 days and the 30-days morality rate was 2.41%, which was more than that in the SBRT group [23]. Our results are consistent with the findings.

This study is limited by the short follow-up period of SBRT patients, which affected their survival rates. Because of this, OS may have favored SBRT. Longer follow-up is essential for adequate comparison of these groups and is ongoing. Another limitation is that fewer patients experienced pretreatment surgical staging in the SBRT group. The clinical staging of patients with NSCLC guidelines was often demonstrated as a poor predictive value by previous reports [24]. To improve the accuracy of clinical staging, PET scan is considered as an important tool. However, in our study, only a minority of patients had undergone FDG-PET scans. Notably, adjuvant systemic therapy (including chemotherapy and EGFR-TKI) was barely used in both the lobectomy and SBRT cohorts, because the patients were too old or too weak to tolerate it. Therefore, systemic therapy made a modest contribution to OS difference between the lobectomy and SBRT groups.

## Conclusion

After matching with PSM for age, T stage, and other factors, the outcomes of LRFS, RFS, OS and CSS were not different between the two groups. SBRT offers a low-risk effective alternative for high-risk patients. However, treatment of these very high-risk patients with stage I NSCLC remains a challenge. Outcomes from findings in both treatment cohorts are strongly influenced by comorbidity and risk. Only randomized comparisons between lobectomy and SBRT will avoid the imbalances and selection biases present in this and other nonrandomized comparisons.

## Abbreviations

CSS: Cancer-specific survival
CT: Computed tomography
DM: Distant metastasis
DMRFS: Distant metastasis recurrence-free survival
EGFR-TKI: Epidermal growth factor receptor tyrosine kinase inhibitors
FDG-PET: Fluorodeoxyglucose -positron emission tomography
FEV1: Forced expiratory volume in 1 s
GTV: Gross tumor volume
ISABR: Immuno Stereotactic Ablative Body Radiotherapy
ITV: Internal target volume
LR: Local recurrence
LRFS: Local recurrence-free survival
NSCLC: Non-small cell lung cancer
OS: Overall survival
PS: Performance status
PSM: Propensity score matching
PTV: Planning target volume
RFS: Recurrence-free survival
RR: Regional recurrence
RRFS: Regional recurrence-free survival
SBRT: Stereotactic body radiation therapy
